# Identifying the Socioeconomic, Demographic, and Political Determinants of Social Mobility and Their Effects on COVID-19 Cases and Deaths: Evidence From US Counties

**DOI:** 10.2196/31813

**Published:** 2022-03-03

**Authors:** Niloofar Jalali, N Ken Tran, Anindya Sen, Plinio Pelegrini Morita

**Affiliations:** 1 School of Public Health and Health Systems Faculty of Applied Health Sciences University of Waterloo Waterloo, ON Canada; 2 School of Public Health and Health Systems University of Waterloo Waterloo, ON Canada; 3 Department of Economics University of Waterloo Waterloo, ON Canada; 4 Department of Systems Design Engineering University of Waterloo Waterloo, ON Canada; 5 JW Graham Information Technology Emerging Leader Chair Applied Health Informatics University of Waterloo Waterloo, ON Canada; 6 Institute of Health Policy Management and Evaluation University of Toronto Toronto, ON Canada

**Keywords:** COVID-19, cases, deaths, mobility, Google mobility data, clustering

## Abstract

**Background:**

The spread of COVID-19 at the local level is significantly impacted by population mobility. The U.S. has had extremely high per capita COVID-19 case and death rates. Efficient nonpharmaceutical interventions to control the spread of COVID-19 depend on our understanding of the determinants of public mobility.

**Objective:**

This study used publicly available Google data and machine learning to investigate population mobility across a sample of US counties. Statistical analysis was used to examine the socioeconomic, demographic, and political determinants of mobility and the corresponding patterns of per capita COVID-19 case and death rates.

**Methods:**

Daily Google population mobility data for 1085 US counties from March 1 to December 31, 2020, were clustered based on differences in mobility patterns using K-means clustering methods. Social mobility indicators (retail, grocery and pharmacy, workplace, and residence) were compared across clusters. Statistical differences in socioeconomic, demographic, and political variables between clusters were explored to identify determinants of mobility. Clusters were matched with daily per capita COVID-19 cases and deaths.

**Results:**

Our results grouped US counties into 4 Google mobility clusters. Clusters with more population mobility had a higher percentage of the population aged 65 years and over, a greater population share of Whites with less than high school and college education, a larger percentage of the population with less than a college education, a lower percentage of the population using public transit to work, and a smaller share of voters who voted for Clinton during the 2016 presidential election. Furthermore, clusters with greater population mobility experienced a sharp increase in per capita COVID-19 case and death rates from November to December 2020.

**Conclusions:**

Republican-leaning counties that are characterized by certain demographic characteristics had higher increases in social mobility and ultimately experienced a more significant incidence of COVID-19 during the latter part of 2020.

## Introduction

In March 2020, COVID-19 was acknowledged by the World Health Organization (WHO) to be a global pandemic [1]. Since then, governments worldwide have implemented a series of lockdown measures intended to reduce the spread of the disease. The efficacy of these measures, in the absence of a vaccine or effective therapy, has varied across countries. Initial evidence on lockdown measures implemented in China suggested that reducing interpersonal physical contact or reducing the movement of the population is an effective means to control the spread of the virus [2]. These findings spurred national and subnational policies restricting population mobility, including social distancing (physical distancing between people who are not from the same household) [3] and stay-at-home (SAH) or shelter-in-place (SIP) orders, which required people to stay at home except for essential activities [4,5].

In addition to the direct impacts of such policies, evaluating the effects of demographic and socioeconomic factors on population mobility is also important as there were non-pandemic-related events that significantly impacted public movements in the U.S. after the first wave of the pandemic. Specifically, the summer of 2020 witnessed many demonstrations and public rallies in the U.S. in response to a series of events, including the death of George Floyd. Social distancing receded into the background despite rising caseloads and deaths due to COVID-19. The initial decline in public movement that occurred during the early months of the pandemic was succeeded by rapid increases in social mobility through much of the U.S. [6]. Increases in social mobility also occurred as many jurisdictions modified their SAH orders, allowed more businesses to reopen, and relaxed rules on social distancing [7]. This rise in mobility has been linked to higher COVID-19 cases in these regions [8]. Public mobility may have also increased during fall 2020 because of public rallies and social gatherings associated with the US presidential election.

A growing amount of research has used mobility data from social media platforms (Google, Twitter, and Facebook) and mobile phone providers to understand changes in mobility during the pandemic [9,10], the relationship between population mobility and the spread of COVID-19 cases [8-18], and the effects of nonpharmaceutical interventions (NPIs) on mobility [5,19,20]. The consensus from these studies is that increased mobility is associated with higher COVID-19 case counts. Badr et al [15] used cell phone data from 25 counties provided by Teralytics and found that reduced mobility patterns are associated with reduced COVID-19 incidence rates. Using mobile phone data from Safegraph, Gao et al [20] similarly found that lower mobility (more time at home) is associated with a reduced spread of COVID-19 across states. Glaeser et al [19] also used Safegraph data and found reduced mobility to be correlated with lower cases for some US cities. Using Google data from different jurisdictions, other studies found a positive correlation between mobility and COVID-19 case counts [11,12,14,17]. These studies are, however, limited; they investigated social mobility across a small number of US counties during the early days of the pandemic. As such, they were unable to capture socioeconomic, demographic, and political determinants of mobility [21-25].

We evaluated the determinants and consequences of population movements in 1089 US counties from the start of the pandemic to December 2020. This study contributes to the literature by using clustering analysis and other tools to evaluate the impacts of different socioeconomic and demographic characteristics on social mobility in a sample of US counties. We also investigated the effects of such mobility decisions on daily per capita COVID-19 cases and deaths. Social mobility was measured through the use of Google mobility indicators at retail and recreational venues, grocery and pharmacy stores, workplaces, and residences. Robust statistical findings based on such analysis would inform policymakers in crafting efficient and effective NPIs that could curb the spread of COVID-19.

Our results demonstrate that clusters with higher mobility at retail outlets, grocery and pharmacy stores, and workplaces and a lower duration of stay at residences also have a higher percentage of population aged 65 years and over, a larger population share of Whites with less than high school and college education, a higher percentage of the population with less than a college education, a lower percentage of the population using public transit to work, and a smaller share of voters who voted for Clinton during the 2016 presidential election relative to other clusters. The clusters with higher mobility also experienced pronounced increases in per capita COVID-19 daily case and death rates from November to December 2020. These findings are consistent with other studies that suggest that Trump-leaning counties experienced increases in social mobility and less stringent policies after the first wave of the pandemic, which was succeeded by higher levels of disease severity during the latter months of 2020.

## Methods

### Data

#### COVID-19 Incidence Data

**Figure 1:**
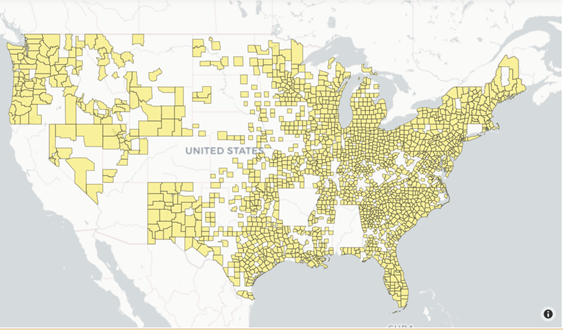
Geographic distribution of counties.

The daily numbers of confirmed cases and deaths due to COVID-19 at the county level were downloaded from the Center for Systems Science and Engineering (CSSE) at Johns Hopkins University (JHU) [26]. For the 1089 counties in our sample, the mean (SD) of confirmed cases and deaths (both per 100,000 of population) were 1541.27 (1905.59) and 33.72 (44.78), respectively. Figure 1 reveals the distribution of counties in our sample. There is a significant concentration of counties in the East, Northeast, and certain southern states. There are fewer counties from the midwestern and southwestern parts of the United States. This is because Google mobility data (discussed later) are less available for counties with lower population density. This is a limitation of our analysis.

#### Population Mobility

Data on population mobility were obtained from Google’s COVID-19 *Community Mobility Reports*. Google creates social mobility data from users who have turned on the Location History setting of Google accounts on their phones and have agreed to share this information. Google mobility indicators are with respect to population-level daily visits to grocery and pharmacy stores, which include grocery markets, food warehouses, farmers’ markets, specialty food shops, drug stores, and pharmacies; parks, which consist of local parks, national parks, public beaches, marinas, dog parks, plazas, and public gardens; transit stations, comprising subway, bus, and train stations; retail stores and recreation outlets consisting of places such as restaurants, cafes, shopping centers, theme parks, museums, libraries, and movie theaters; and workplaces. The Google mobility data also provide an index on the duration of stay at residences. Google mobility indicators for transit hubs and parks were omitted because of large numbers of missing values for the counties included in this study.

A prepandemic baseline mobility value was determined using the median mobility for each day of the week from January 3 to February 6, 2020 [27]. Subsequent mobility values were normalized to baseline. Counties with missing values less than or equal to 10% for each indicator were selected for the study. Missing values were replaced by the average from 3 prior days. The availability of Google data determined which counties we used in our analysis. The final data set contained observations for 1089 counties, which is roughly 35% of the total number of counties (N=3142) in the United States. Daily values were available for the first and second waves of the pandemic from March 11 to December 31, 2020.

With the exception of the residential index, daily values for each index were calculated relative to baseline, which was defined as the median for the corresponding day of the week, during the 5-week period from January 3 to February 6, 2020. Hence, each daily value is the percentage change in the social mobility category relative to its baseline, which shows how the number of visits to different destinations in a day have changed in percentage terms since the onset of the pandemic. The Google residential index represents the duration of stay at an individual’s residence relative to the 5-week baseline. The values in this index are the percentage differences in time spent at home relative to the baseline period.

#### County-Level Socioeconomic, Demographic, and Political Data

The 2016 census data were collected by the Massachusetts Institute of Technology (MIT) Election Data and Science Lab [18]. These data were supplemented by county variables collected by other studies [23,25]. To validate that our samples were representative of all US counties, we compiled summary statistics of socioeconomic and demographic variables between our sample and all counties (Table 1). In summary, there did not seem to be significant differences in most variables between all counties and our sample. The exception is population, where our sample mean was more than 2.5 times that of the mean for all counties. In a similar vein, although all counties have 58% of the population in rural areas, the corresponding statistic for our sample was only approximately 31%. These discrepancies can be explained by the fact that Google's social mobility indicators are only available for counties with larger populations that are more densely populated. This is consistent with the visualization of counties in our sample from Figure 1.

**Table 1:** Sample statistics of census variables for all counties and our sample based on daily values.

Variable			All counties				Our sample		
		Mean (SD)		Minimum	Maximum	Mean (SD)		Minimum	Maximum
**Politics**									
	Population voting for Trump in 2016 (%)	28.13 (8.44)		1.93	76.32	24.28 (7.22)		2.63	66.42
	Population voting for Clinton in 2016 (%)	14.07 (7.41)		0	49.02	17.18 (7.33)		2.73	42.86
	Registered voters as population (%)	74.86 (5.31)		43.14	95.08	73.49 (5.14)		47.33	90.63
**Demographics**									
	Whites (%)	77.36 (19.74)		0.76	100.00	73.57 (18.63)		2.78	97.34
	African Americans (%)	8.96 (14.5)		0	86.19	9.96 (12.21)		0.09	76.55
	Hispanics (%)	8.99 (13.66)		0	98.96	11.03 (13.41)		0.68	95.48
	Foreign born (%)	4.62 (5.63)		0	52.23	7.12 (6.81)		0.40	52.23
	Females (%)	49.98 (2.33)		21.51	58.50	50.62 (1.30)		38.76	56.03
	Population aged 29 years and under (%)	37.34 (5.44)		11.84	70.98	39.24 (4.98)		13.64	61.69
	Population aged 65 years and older (%)	17.63 (4.44)		3.86	53.11	15.57 (3.93)		6.95	53.11
	Less than high school education (%)	14.23 (6.54)		1.28	51.48	12.44 (5.26)		2.08	41.34
	Less than college education (%)	79.22 (9.14)		19.79	97.02	73.98 (10.11)		26.34	90.86
	Whites with less than high school education (%)	11.04 (5.33)		0	41.76	9.11 (3.92)		0.97	25.57
	Whites with less than college education (%)	77.00 (10.36)		9.19	95.92	71.28 (11.58)		15.30	89.96
**Socioeconomics**									
	Median household income (US $)	47,817.60 (12482.4)		18,972.00	125,672.00	53,798.50 (13905.9)		28,452.00	125,672.00
	Rural population (%)	58.48 (31.45)		0	100.00	31.733 (22.08)		0	100.00
	Population density (number of people per square mile)	582.71 (3761.83)		0.26	179,922.30	1397.32 (6127.90)		6.22	179,922.30
	Hospitals per 100,000 of population (number of hospitals per 100,000 of population)	0.61 (0.94)		0	10.56	0.25 (0.166)		0	1.61
	Poverty rate (%)	15.16 (6.07)		2.60	48.40	13.35 (4.87)		2.60	37.30
	Population without health insurance (%)	0.09 (0.05)		0.01	1.62	0.09 (0.06)		0.02	1.62
	Share of population using public transit for commuting to work (%)	0 (0.01)		0	0.26	0.01 (0.02)		0	0.26

### Clustering

**Figure 2:**
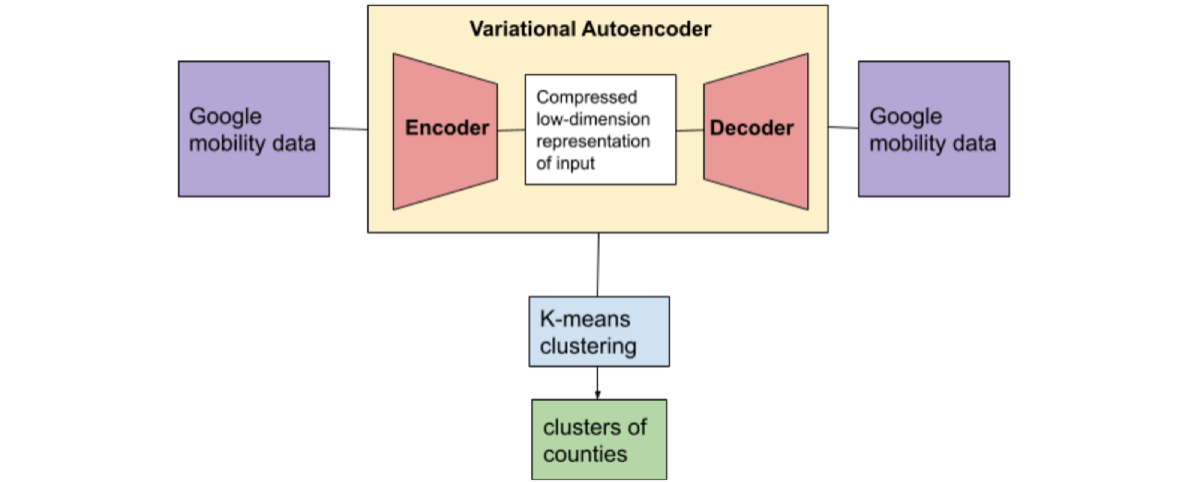
Methodology for identifying different clusters of counties using a variational autoencoder.

Figure 2 summarizes our methodology for identifying different clusters of counties using Google mobility indicators. Clustering is an unsupervised learning technique that partitions a data set into groups or clusters based on similarity measures. This study leveraged partitioning-based algorithms, which divided the data set into partitions, where each partition was a cluster. For each county included in this study, data were clustered based on a combination of the daily values of the 4 Google mobility indicators. To identify the different clusters of counties, we performed 2 steps [28]:

1. Compressing the multidimensional time series data to extract the latent variables using deep neural networks
2. Using K-means clustering to identify the different clusters of counties based on latent variables’ representations

To compress the multidimensional time series, we implemented the variational autoencoder (VAE) architecture based on long short-term memory (LSTM) [29-31]. The principal concept of this generative approach is to project high-dimensional data into latent variables. Our model comprised 4 blocks [32]:

1. Encoder: Defined by the LSTM layers, the multidimensional time series input (x) are fed into the LSTM.
2. Encoder to latent layer: Defined by a linear layer, which identifies the mean and SDs of the last hidden layer of the encoder. During the training process, the multigaussian distributions are defined and reparametrized iteratively by the mean and SDs derived from latent vectors.
3. Latent layer to decoder layer: The latent variables (z) are sampled from the distribution and pass through a linear layer to identify the decoder input.
4. Decoder: Defined by the LSTM layers, which uses latent variables (z) to reconstruct the original data [33].

Identifying the true posterior distribution is intractable [33]. Therefore, to construct the original data, the probabilistic encoder model was approximated by normal distribution p(z|x)N(0,1) and used as a probability decoder [30,33]. Hence, the reconstruction of input was defined by sampling from the distribution of latent variables (z).

To evaluate the performance of the model, the loss function was defined as follows:

- The divergence from the approximated distribution and the true distribution

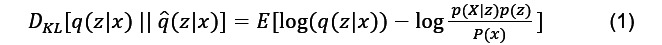







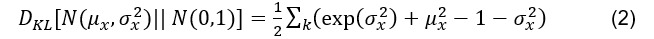

- The mean squared error loss calculated the difference between original and reconstructed input data

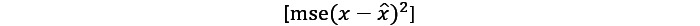

- The total loss is defined as sum of 2 losses:

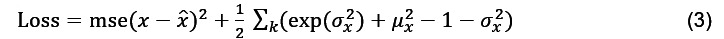




The model was trained in Python 3.6 using the Keras library [34] with the Adam optimizer. The batch size and number of the epochs were set to 10 and 100, respectively. The number of nodes for encoder and decoder hidden layers was set to 500. The dimensionality of latent variables was set to 3. We also implemented the L1 and L2 regularizers to avoid overfitting. To evaluate the performance of the model, the VAE total loss was used to identify the reconstruction error between encoder input and decoder output.

Once the model was trained and the encoder, decoder, and VAE were constructed, the output of the encoder model was selected as the representation of the multidimensional patterns of each county. K-means clustering was used to identify the similar segmentation of the counties. To identify the optimum number of clusters as well as the homogeneity of data points within each cluster, the elbow method [35] and the silhouette score [36] were used.

### Explaining the Socioeconomic Characteristics of Similar Counties

**Figure 3:**
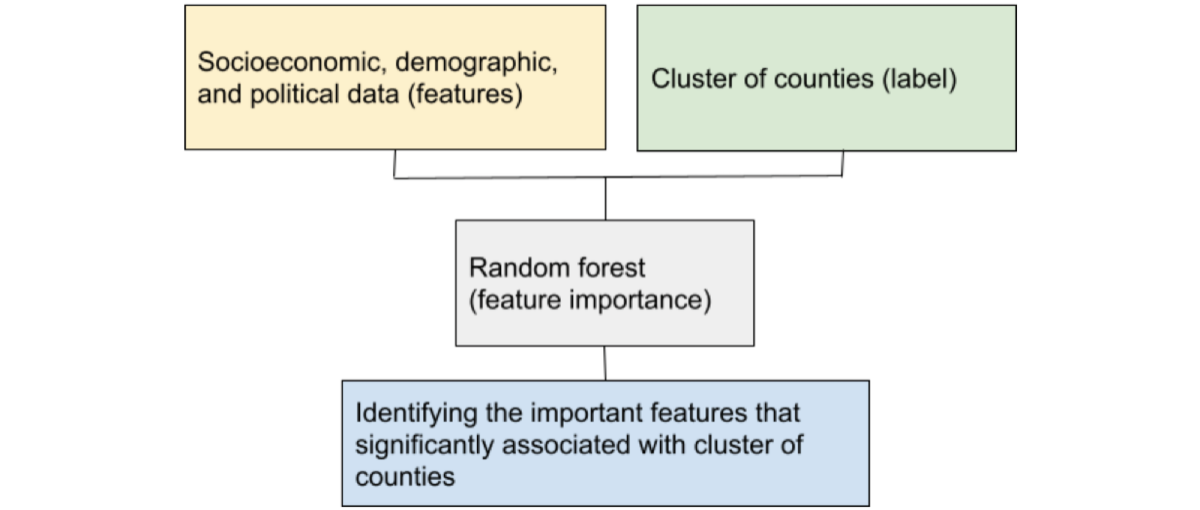
Framework to identify the socioeconomic characteristics of different clusters of counties using random forest feature importance.

To compare the socioeconomic characteristics of the counties in each cluster, the 2016 MIT election data were used as input, while the classes were the cluster labels. The data were divided into training and testing sets with a 70:30 split, respectively. The random forest classifier [37] with 10 k-fold cross-validations was used to build the predictive models. The area under the curve (AUC) of the model was calculated, and the most important features associated with the cluster numbers were defined as the parameters describing the characteristics of counties in each cluster. Feature scores of different census variables for the clusters were computed, which yielded an idea of the relative importance of different socioeconomic and demographic factors for explaining the different clusters. Figure 3 summarizes our approach.

## Results

### Clustering

**Figure 4:**
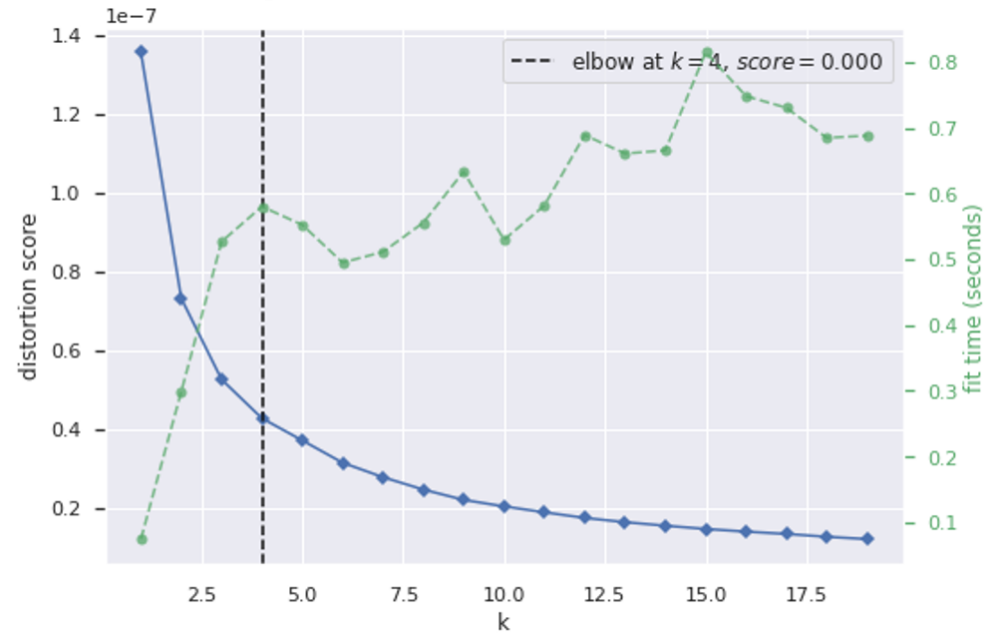
Distortion score elbow for K-means clustering.

This study leveraged a partitioning-based deep learning model to cluster counties based on similarities in social mobility. For each county included in this study, data were clustered based on a combination of the daily values of the 4 Google mobility indicators (retail, grocery and pharmacy, workplace, and residence). The multidimensional time series of Google social mobility indicators from 1089 counties was divided into training and testing sets and fed into the VAE model. The result demonstrated a loss of 0.08. The latent variables were extracted as the output of the encoder. The K-means clustering algorithm identified 4 social mobility clusters. The number of counties in these clusters, which were termed as 0, 1, 2, and 3, were 215, 338, 473, and 59, respectively. Figure 4 gives the distortion scores of the K-means clustering.

### Google Social Mobility Trends

**Figure 5:**
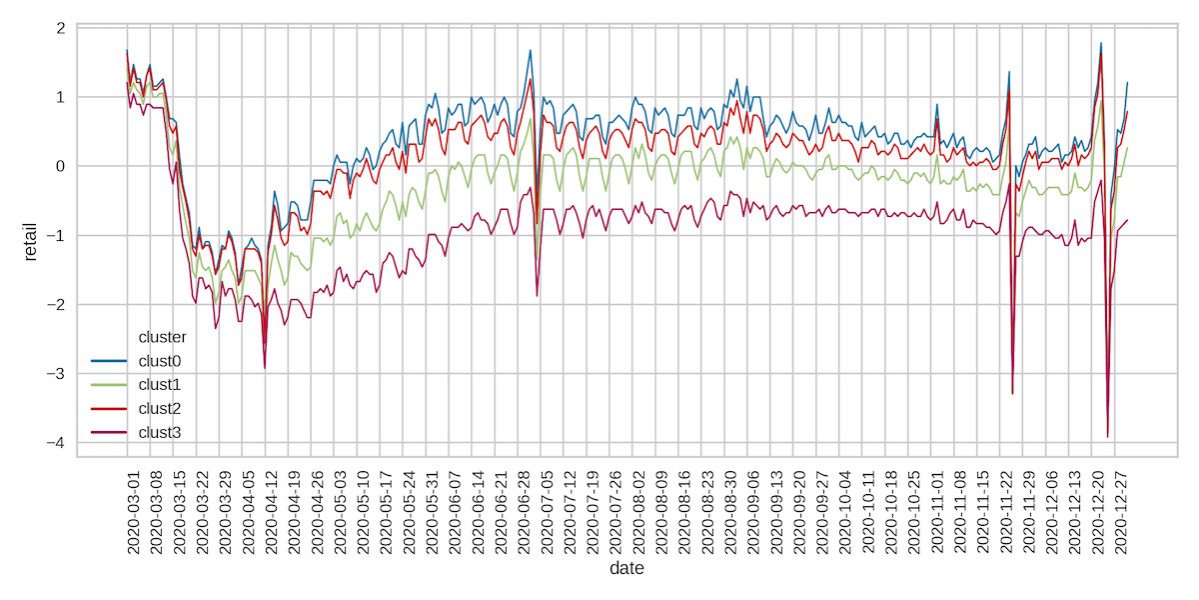
Google retail mobility.

**Figure 6:**
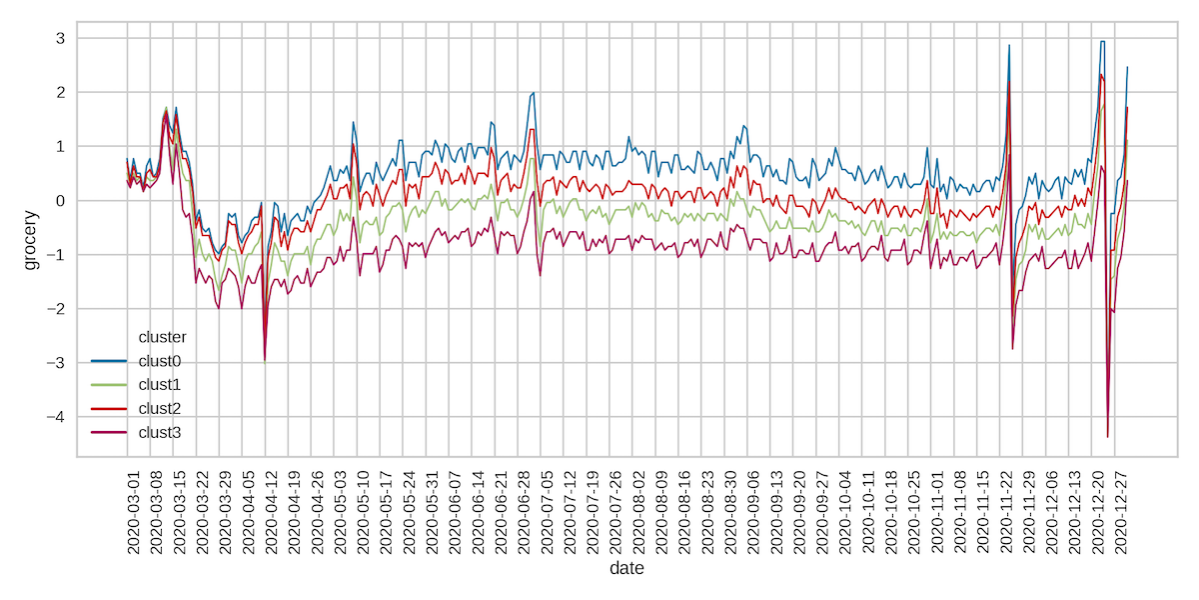
Google grocery and pharmacy mobility.

**Figure 7:**
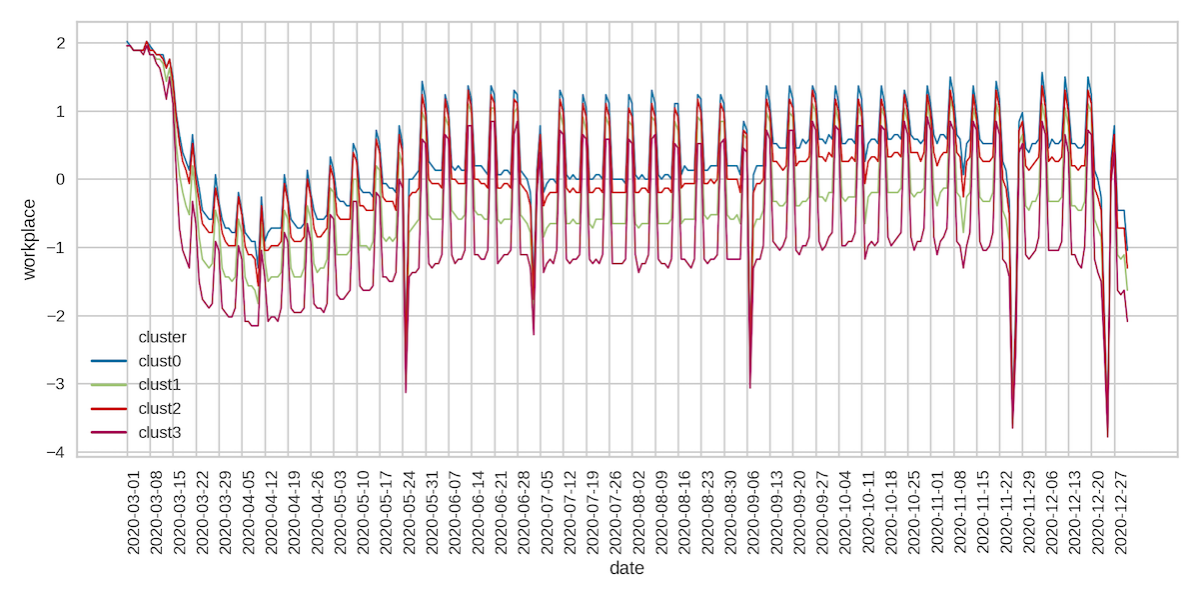
Google workplace mobility.

Across all clusters, visits to retail stores fell significantly after the start of the pandemic until around mid-April, followed by a steady increase and plateauing in early July (Figure 5). Visits to retail outlets began to decline again in late September but then began an upward trend starting on Thanksgiving weekend until the end of December. Retail social mobility values were the highest for cluster 0, followed by clusters 2 and 1, with cluster 3 having the lowest social mobility. Grocery and pharmacy mobility trends reflected those seen for retail social movements but were less pronounced (Figure 6). Cluster 0 had the highest values of grocery mobility, followed by clusters 2, 1, and 3. Workplace mobility showed an initial decline at the start of the pandemic, followed by a steady increase from early May onward (Figure 7). Spikes in mobility were observed during the weekend, which did not significantly decline relative to prepandemic observations. County clusters followed the same order, with cluster 0 having the greatest mobility, followed by clusters 2, 1, and 3.

**Figure 8:**
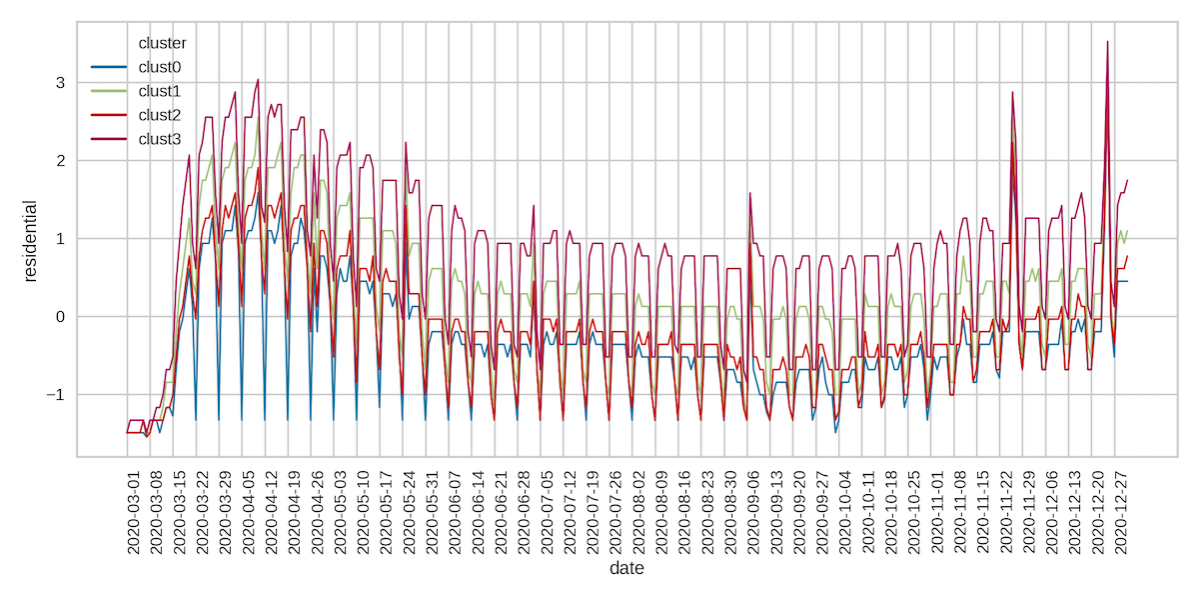
Residential mobility.

Finally, residential mobility followed a reverse pattern relative to the other indicators, with cluster 3 having the highest mobility, followed by clusters 1, 2, and 0 (Figure 8). Residential mobility was highest during the onset of the pandemic, followed by a decreasing trend during spring and summer. From late September onward, residential mobility began to increase, and this trend continued until the end of the sample period. The spikes in mobility captured the weekend effects. Our social mobility data indicated differences in mobility between clusters, with counties in cluster 0 having the highest retail, grocery, and workplace mobility and the lowest residential mobility. In contrast, counties in cluster 3 had the lowest social mobility and the highest residential mobility.

### Relationship Between Google Social Mobility Indicators and County Characteristics

To determine whether county characteristics are correlated with differences in social mobility between the clusters, we obtained socioeconomic, demographic, and political data from each county from 2016 census data [18]. These data included 2016 election returns, race, median income, total population, percentage of rural areas, and education level of the population for age and race. These data were supplemented by county variables collected by other studies [23,25].

A random forest classifier was used to generate feature scores of different socioeconomic and demographic characteristics of the counties included in each cluster, across all 4 clusters (mean receiver operating characteristic [ROC] AUC 0.871). Table 2 contains the feature scores of all county-level variables.

The top 10 variables in terms of feature scores were percentage of the population aged 65 years and over (0.41715), percentage of females (0.08784), percentage of Whites (0.03869), percentage of Whites with less than college education (0.03772), percentage of Hispanics (0.03369), percentage of Whites with less than high school education (0.03178), percentage of the population using public transit (0.02967), county unemployment rate (0.02759), proportion of voters for Clinton in 2016 (0.02737), and percentage of the population with less than high school population (0.02719). Hence, although political preference and population composition were important, it is important to note the significance of 3 educational variables among the top 10, with the percentage of the population with less than college education being the 11th variable in terms of feature score.

To explore the top 11 socioeconomic, demographic, and political variables impacting social mobility further, we determined the mean population percentage for each county-level variable across clusters (Table 3). The table also contains results of statistical tests of significance of sample means between clusters. The Z test of sample means was performed to compare the significance of different county-level variables for different clusters. Results demonstrated several variable similarities for clusters with the highest social mobility. The percentage of the population aged 65 years and over, Whites, the percentage of whites with less than high school and college education, and the percentage of the overall population with less than college education were higher in counties defined by clusters 0 and 2. Tests of equality of sample proportions and means confirmed that there was a statistically significant difference between clusters 0 and 2 versus clusters 1 and 3 for these population variables. In contrast, the percentage of Hispanics, percentage of the population using public transit for work, and percentage voting for Clinton in 2016 were lower in clusters 0 and 2 relative to clusters 1 and 3. There was no consistent, significant difference across clusters for the percentage of females, population with less than high school education, and unemployment rates.

**Table 2:** Feature scores of county-level variables.

Feature	Score
Percentage aged 65 years and older	0.41715
Percentage of females	0.08784
Percentage of Whites	0.03869
Percentage of Whites with less than college education	0.03772
Percentage of Hispanics	0.03369
Percentage of Whites with less than high school education	0.03178
Percentage of population using public transit for commuting to work	0.02967
Unemployment rate	0.02759
Percentage voting for Clinton in 2016	0.02737
Percentage with less than high school education	0.02719
Percentage with less than college education	0.02429
Hospitals per 100,000 of population	0.02385
Percentage of rural population	0.0221
Population density	0.02178
Percentage of foreign born	0.02118
Poverty rate	0.02051
Percent without health insurance	0.02003
Percentage voting for Trump in 2016	0.01992
Median household income	0.01911
Percentage aged under 29 years	0.01852
Registered voters as a percentage of population	0.01682
Percentage of African Americans	0.01319

**Table 3:** Differences in county-level variables across clusters.

Variable	Sample mean (%)					*P* value of sample means between clusters			
	Cluster 0	Cluster 1	Cluster 2	Cluster 3	Clusters 0 and 1		Clusters 0 and 3	Clusters 1 and 2	Clusters 2 and 3
Population aged 65 years and older	17.10	14.20	16.20	13.00	<.01		<.01	<.01	<.01
Females	50.40	50.70	50.70	50.40	.01		.99	.99	.23
White	81.50	66.30	77.10	58.60	<.01		<.01	<.01	<.01
Whites with less than college education	78.20	65.00	75.20	51.10	<.01		<.01	<.01	<.01
Hispanics	6.90	15.50	8.20	19.80	<.01		<.01	<.01	<.01
Whites with less than high school education	11.20	7.10	10.10	5.10	<.01		<.01	<.01	<.01
Population using public transit for commuting to work	0.30	1.20	0.30	3.70	<.01		<.01	<.01	<.01
Unemployment rate	7.50	7.20	7.40	6.30	.06		<.01	.06	<.01
Voting for Clinton in 2016	13.80	20.10	15.30	27.20	<.01		<.01	<.01	<.01
Less than high school education	13.50	11.70	12.60	11.50	<.01		.02	.01	.17
Less than college education	79.50	69.20	76.90	58.50	<.01		<.01	<.01	<.01

### Trends in Daily Cases/Deaths by Cluster

**Figure 9:**
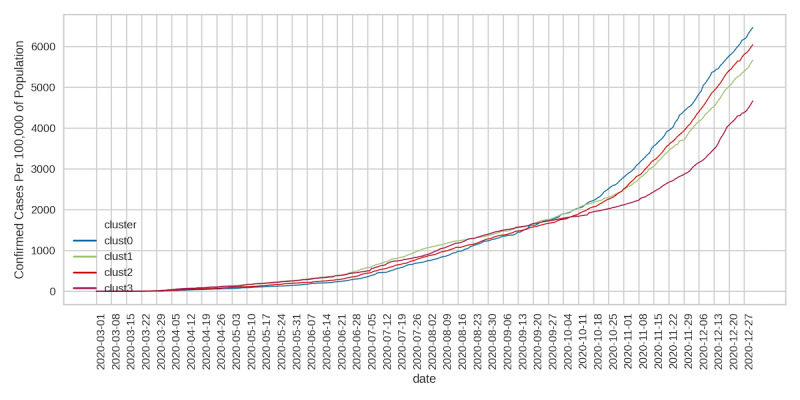
Daily cases per 100,000 residents.

**Figure 10:**
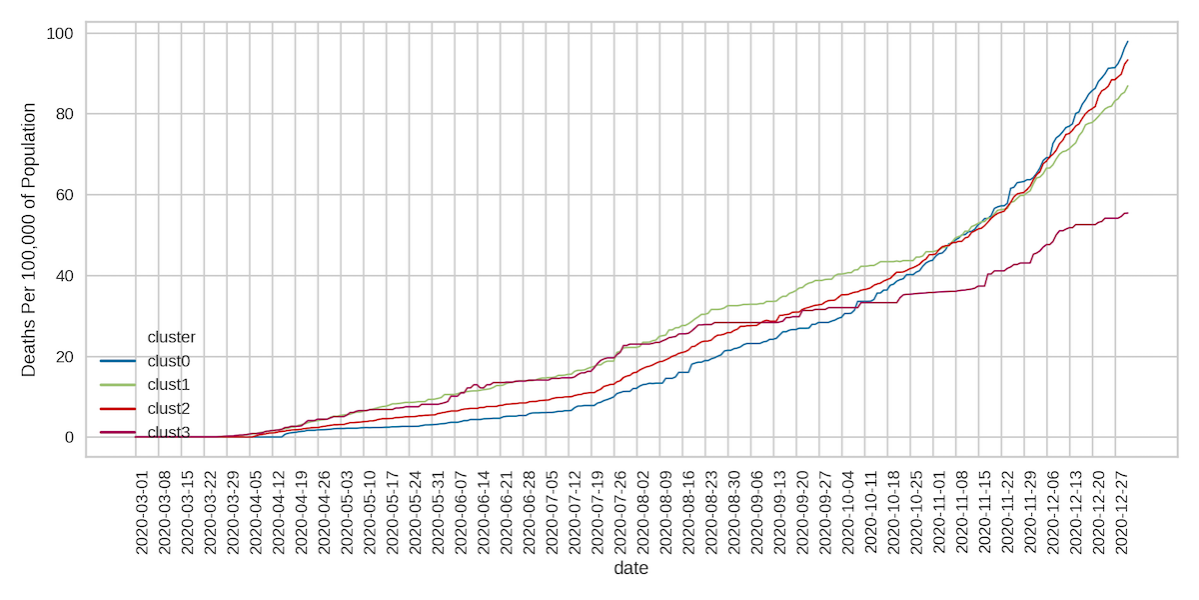
Daily deaths per 100,000 residents.

Given that policies restricting population mobility were established to curb the spread of COVID-19, we sought to determine whether county clusters with higher social mobility indicators (clusters 0 and 2) reported elevated viral cases and deaths. The daily number of confirmed cases and deaths due to COVID-19 at the county level was obtained from the CSSE at the JHU. We determined the median daily per capita cases (Figure 9) and deaths (Figure 10) by cluster. During the first months of the pandemic, per capita daily cases were quite comparable across clusters (Figure 9). There was a visible divergence that occurred at the beginning of October (onset of the second pandemic wave), with daily cases rising sharply in clusters 0, 1, and 2 relative to cluster 3. For the remainder of the period examined, cluster 0 had the highest number of daily cases, followed by clusters 2 and 1. Cluster 3 retained relatively lower daily cases. Interestingly, clusters 0 and 2 had lower daily deaths until the beginning of September (Figure 10). Daily deaths in these clusters then increased rapidly, and by the beginning of October, per capita deaths in clusters 0, 1, and 2 were higher than in cluster 3.

## Discussion

### Principal Findings

This study aimed to assess the effect of county-level characteristics on population mobility and the consequences of this mobility on the spread of COVID-19. To the best of our knowledge, this is the first study that has used unsupervised machine learning to understand differences in population mobility across US counties during the first and second waves of the pandemic and determine the relative importance of a wide array of socioeconomic, demographic, and political variables in defining different mobility-based clusters.

Our results demonstrate that of the 4 clusters defined by Google social mobility indicators, the clusters with higher retail, grocery, and work mobility (and lower residential mobility) had several similar population characteristics. Specifically, counties with greater social mobility also had a higher percentage of the population aged 65 years and over, Whites with less than high school and college education, and overall population with less than college education. Counties in these 2 clusters also had a lower share of the population that is Hispanic, the percentage of the population using public transit to work, and the share of voters who voted for Clinton during the 2016 presidential election. Research does suggest that Whites with less than college education constituted a significant voting block for Trump during the 2016 election [38]. In line with this, the 2 clusters with the greatest social mobility also experienced higher per capita COVID-19 case and death rates during most of November and December 2020. These results are consistent with Xie and Li [39], who also used county-level data during the early days of the pandemic and found lower education levels to be correlated with higher infection rates.

The significant increase in COVID-19 cases and deaths in clusters 0 and 2 during November and December 2020 could be a consequence of public rallies and general disregard for social distancing and safety protocols by pro-Trump voters [40]. Although we cannot prove this, the majority of counties in these clusters were Republican leaning during the 2016 presidential election. Moreover, our finding of higher per capita daily COVID-19 cases and deaths in such counties is consistent with other studies. Desmet and Wacziarg [41] found that early on during the pandemic, Republican counties actually experienced lower COVID-19 cases and therefore had lax attitudes toward mask wearing, social distancing, and lockdown measures. However, as the pandemic spread to Trump-leaning counties, population preferences for less stringent social distancing policies had already been formed, making it difficult for policymakers to implement stricter restrictions on social mobility. As a result, this led to greater disease severity in Trump-leaning counties. In a similar vein, Allcott et al [42] found that areas with more Republicans engaged in less social distancing, controlling for other factors, including public policies. In summary, these findings corroborate our own results. Social mobility in the aftermath of the first wave of the pandemic was much higher in Republican counties, which ultimately resulted in higher COVID-19 cases and associated deaths relative to other counties that were Democrat leaning.

Social media is increasingly being used to capture population movements and understand their corresponding impacts on COVID-19 incidence. Social media–based data, including those presented here, have some limitations. Specifically, there is the possibility of sample selection bias if Google Maps users have specific demographic characteristics and are not distributed uniformly across the population. However, data from Statista indicate that in the U.S., Google Maps had 154 million users in April 2018 [43]. Further, published research has done a comparison of Google mobility data against corresponding cellular-generated information by other providers and has found a close correspondence. Specifically, Szocska et al [44] constructed a mobility index and an SAH/resting index based on data on almost all phone subscribers in Hungary and found a close correlation with corresponding Google mobility indices at the national level. There are also a significant number of published studies that have used Google mobility data to capture population movements for different countries and have found them to be important in predicting movements in COVID-19 (Bryant and Elofsson [11], Askitas et al [45], and Stevens et al [46]). For these reasons, we think there is a high likelihood that Google mobility data do reflect population movements. However, Google mobility data do not include information on certain types of public movements, such as election rallies or community gatherings.

Our research demonstrates the usefulness of publicly available Google mobility data and unsupervised machine learning methods in establishing relationships between county-level characteristics, mobility decisions, and COVID-19 incidence. These findings have important implications for policymakers and public health officials in understanding the effects of NPIs, as the efficacy of such measures on mobility is influenced by underlying socioeconomic, demographic, and political ideology characteristics. The use of Google data enables researchers to assess the types of public movements that are most contributory to COVID-19 spread.

The results of this study provide a unique lens on the potential of machine learning to understand social mobility behaviors. These findings are critical for public health organizations trying to understand the levels of mobility in their counties, in addition to providing insights into some of the underlying factors (ie, social determinants of health) contributing to regional differences in COVID-19 caseloads.

### Conclusion

Our results emphasize a role for machine learning methods in public health. Publicly available Google data, in conjunction with census data, can be used to understand the socioeconomic, demographic, and political determinants driving population mobility choices across US counties. This knowledge can assist policymakers in developing NPIs to restrict viral spread during the COVID-19 pandemic.

## Abbreviations

AUC: area under the curve
CSSE: Center for Systems Science and Engineering
JHU: Johns Hopkins University
LSTM: long short-term memory
MIT: Massachusetts Institute of Technology
NPI: nonpharmaceutical intervention
SAH: stay-at-home
SIP: shelter-in-place
VAE: variational autoencoder
